# Free Fatty Acids Correlate with the Interleukin-1 β and Interleukin-1 Receptor Antagonist in the Early Subacute Phase of Stroke

**DOI:** 10.3390/biom15111537

**Published:** 2025-10-31

**Authors:** Dariusz Kotlega, Arleta Drozd, Agnieszka Zembron-Lacny, Barbara Morawin, Karina Ryterska, Malgorzata Szczuko

**Affiliations:** 1 Department of Pharmacology and Toxicology, University of Zielona Gora, 65-417 Zielona Gora, Poland; 2 Northampton General Hospital NHS Trust, University Hospitals of Northamptonshire, Northampton NN1 5BD, UK; 3 Department of Applied Microbiology and Human Nutrition Physiology, Faculty of Food Sciences and Fisheries, West Pomeranian University of Technology, 71-310 Szczecin, Poland; 4 Faculty of Medicine, University of Warsaw, 02-089 Warsaw, Poland; a.zembron-lacny@cm.uz.zgora.pl; 5 Department of Applied and Clinical Physiology, University of Zielona Gora, 65-417 Zielona Gora, Poland; b.morawin@cm.uz.zgora.pl; 6 Department of Human Nutrition and Metabolomics, Pomeranian Medical University in Szczecin, 71-460 Szczecin, Poland; 7 Department of Bromatology and Nutritional Diagnostics, Pomeranian Medical University in Szczecin, 71-460 Szczecin, Poland; malgorzata.szczuko@pum.edu.pl

**Keywords:** ischaemic stroke, free fatty acids (FFAs), interleukin-1 beta (IL-1β), interleukin-1 receptor antagonist (IL-1Ra), inflammation

## Abstract

Inflammation contributes to the pathogenesis of ischaemic stroke both as a long-term causal factor and through the inflammatory cascade in acute stroke. Interleukin-1 beta (IL-1β) is a potent pro-inflammatory molecule, while interleukin-1 receptor antagonist (IL-1Ra) acts as its antagonist. Free fatty acids (FFAs) play a role in atherosclerosis formation and serve as substrates for inflammatory molecules. This study aimed to determine the potential interplay between FFAs, IL-1β, and IL-1Ra in stroke patients. A prospective analysis was conducted on 73 ischaemic stroke patients. All participants had their FFA, IL-1β, and IL-1Ra levels assessed. Significant correlations between IL-1β and certain FFAs were detected: C15:0 pentadecanoic acid (rho = 0.488), C15:1 cis-10 pentadecanoic acid (rho = 0.473), C17:1 cis-10 heptadecanoic acid (rho = 0.411), C18:0 stearic acid (rho = 0.302), C24:0 lignoceric acid (rho = −0.280), C24:1 nervonic acid (rho = −0.276), C18:2n6t linoleic acid (rho = −0.272), C17:0 heptadecanoic acid (rho = 0.241), and C13:0 tridecanoic acid (rho = 0.238). After multivariate analysis C15:0 pentadecanoic acid remained statistically significant. The strongest correlation was found between IL-1Ra and fatty acids: C15:1 cis-10-pentadecanoid acid (rho = −0.357), C18:2n6t linoleic acid (rho 0.341) and C24:1 nervonic acid (rho 0.302), but after multivariate analysis significantly correlated remained: C22:1n9 13 erucic acid (rho = 0.299), C18:3n6 gamma-linoleic acid (rho = 0.277), with close to significant correlation with C22:4n6 docosatetraenoate (rho = −0.241, *p* = 0.055). Certain FFAs may play a role in enhancing both pro- and anti-inflammatory responses in the early subacute phase of stroke, where inflammatory and resolving processes are ongoing. Fatty acids such as C15:0 pentadecanoic acid, C15:1 cis-10 pentadecanoic acid and C22:4n6 docosatetraenoate might be involved in pro-inflammatory responses, while C22:1n9 13 erucic acid and C18:3n6 gamma-linoleic acid in the anti-inflammatory pathways with the overlay of IL-1β and IL-1Ra.

## 1. Introduction

Ischaemic stroke is a leading cause of adult disability and a significant contributor to mortality. Most cases are associated with atherosclerosis and thrombotic mechanisms, while less common causes include cardioembolic events and inflammatory processes. Atherosclerosis develops because of chronic inflammation and lipid deposition in the arterial endothelium. In addition to its role in long-term stroke risk, inflammation also plays a critical role in the acute phase of stroke. During stroke, a cascade of inflammatory processes is triggered, involving numerous inflammatory molecules [1].

Interleukin-1 beta (IL-1β) is a member of the IL-1 cytokine family, which plays a key role in inflammation as part of the non-specific innate immune response. IL-1β is the predominant form expressed in the brain and is upregulated following stroke. While IL-1α and IL-1β share receptor interactions, they may exert distinct biological functions [2]. IL-1β has diverse effects, including the induction of cyclooxygenase-2, upregulation of adhesion molecules, and increased production of chemokines, cytokines (IFNγ, TNFα, IL-6), growth factors, matrix metalloproteinases, and nitric oxide. Additionally, IL-1β enhances platelet activation and leukocyte infiltration, promotes angiogenesis, disrupts blood–brain barrier (BBB) integrity, and suppresses neurogenesis. In the acute phase of ischaemic stroke, IL-1β contributes to neuronal injury [3,4]. The primary receptor for IL-1β is IL-1R1, also known as the Toll/interleukin-1 receptor (TIR), which shares homology with Toll-like receptors (TLRs). Beyond its role in neuroinflammation, IL-1β also drives systemic inflammatory responses [5]. It has been implicated in the pathogenesis of type 2 diabetes, though conclusive evidence remains lacking [6]. A randomised clinical trial investigating the monoclonal anti-IL-1β antibody canakinumab in post-myocardial infarction patients demonstrated a reduction in C-reactive protein (CRP) levels by up to 41%, along with a decreased incidence of major cardiovascular events (nonfatal stroke, nonfatal myocardial infarction, and cardiovascular death) [7,8].

Interleukin-1 receptor antagonist (IL-1Ra) prevents IL-1β from binding to its receptor by competitively binding to the type I receptor without triggering activation, thereby blocking the effects of both IL-1α and IL-1β. Knockout models of IL-1Ra exhibit worsened stroke outcomes, including increased infarct size and neuronal death, while IL-1Ra neutralisation exacerbates neuronal injury in experimental models. Endogenous IL-1Ra provides neuroprotection following stroke, primarily through its actions on glial cells [2]. IL-1Ra inhibits the activity of both IL-1α and IL-1β and is clinically approved as the drug anakinra for treating autoimmune disorders such as cryopyrin-associated periodic syndrome (CAPS), Still’s disease, familial Mediterranean fever (FMF), rheumatoid arthritis, and periodic fever syndromes [9,10]. It has also been investigated in stroke patients, where administration of recombinant human IL-1Ra (anakinra) was associated with reductions in white blood cell counts, C-reactive protein (CRP), and IL-6 levels, as well as improved functional outcomes at three months in patients with cortical infarcts. No safety concerns were reported in this study [11].

Free fatty acids (FFAs) can induce the expression of IL-1β, IL-6, and IL-8, an effect that is exacerbated by elevated glucose levels. This pro-inflammatory response is inhibited by IL-1Ra. The inflammatory activity of FFAs is mediated through the docking protein Myeloid Differentiation Factor 88 (MyD88) within the IL-1R/Toll-like receptor (TLR) signalling pathways, specifically TLR2 and TLR4. Among FFAs, oleate, either alone or in combination with palmitate, is predicted to have the strongest effect on IL-1β activation [12]. Several FFAs have been shown to modulate inflammatory mediators. Total n-3 FFAs are associated with lower levels of IL-6, IL-1Ra, and TNFα, along with higher levels of soluble IL-6R, TGFβ, and IL-10. Reduced levels of n-6 FFAs correlate with lower TGFβ and higher IL-1Ra. Lower concentrations of arachidonic acid (AA) and docosahexaenoic acid are linked to increased IL-6 and IL-1Ra levels and decreased TGFβ levels. Similarly, reduced α-linolenic acid levels are associated with elevated CRP and IL-1Ra, while lower eicosapentaenoic acid levels correlate with higher IL-6 and reduced TGFβ. A decrease in docosahexaenoic acid is also linked to lower IL-10 levels [13]. Saturated fatty acids (SFAs) can activate TLR4 and NFκB, leading to the expression of pro-inflammatory cytokines such as RANTES, monocyte chemoattractant protein 1 (MCP-1), inducible nitric oxide synthase (iNOS), IL-12, IL-6, IL-1, and TNFα. Specific SFAs, including palmitate, myristate, and laurate, activate this pathway, whereas n-3 FFAs inhibit it [14,15]. Additionally, total FFA levels have been found to correlate with IL-1β expression [16].

The aim of this study was to investigate the associations between free fatty acids (FFAs) and key innate inflammatory mediators, specifically IL-1β and its antagonist IL-1Ra. Given the advancements in pharmacological therapies targeting inflammatory pathways, we analysed the correlations between these immune system components in the early subacute phase of stroke. Our study presents novel findings that have not been previously reported. These results may contribute to further research into potential therapeutic strategies for stroke, both in prevention and acute-phase management.

## 2. Material and Methods

### 2.1. Subjects

A prospective study was carried out involving 73 patients diagnosed with ischemic stroke, selected based on the inclusion criterion: confirmation of ischemic stroke through clinical symptoms and additional, routine test results, including brain imaging (CT or MRI). Patients with either embolic or atherothrombotic stroke mechanisms were included. Ischemic stroke was defined as a rapid onset of focal or global cerebral dysfunction symptoms lasting 24 h or longer or confirmed via imaging. Exclusion criteria included evidence of intracranial haemorrhage on imaging, active infection symptoms such as a body temperature exceeding 37.4 °C, clinical or biochemical signs of infection, active autoimmune disorders or malignancies, as well as speech or consciousness impairments caused by cerebral, metabolic, or other conditions that could affect the reliability of neuropsychological test results. The patients were admitted to the Neurology Department of a district hospital in Poland, and all were Caucasians. None had been taking omega-3 supplements prior to hospital admission. During their stay, all patients received treatment with statins and acetylsalicylic acid. The control group consisted of 33 non-stroke adults attending the University of Third Age clubs. The control group was older and had greater frequency of dyslipidaemia and diabetes occurrence (age 71.2 ± 4.7, dyslipidaemia n = 10, diabetes n = 7). The frequency of sex distribution, hypertension, ischaemic heart disease and atrial fibrillation was not statistically different between groups.

### 2.2. Free Fatty Acids Analysis

Venous blood samples were obtained on the seventh day following symptom onset (n = 73). Free fatty acid (FFA) analyses were conducted using liquid and gas chromatography (Agilent Technologies 7890A GC System with a SUPELCOWAX 10 Capillary GC Column, Santa Clara, CA, USA) after centrifugation, and the samples were stored at −80 °C. Serum FFAs were converted to methyl esters using a modified Folch method, as previously detailed in other studies [17,18]. The results were expressed as the percentage composition of each fatty acid relative to the total fatty acid content in the analysed samples. The following FFAs were identified in the samples: C13:0 tridecanoic acid, C14:0 myristic acid, C14:1 myristolenic acid, C15:0 pentadecanoid acid, C15:1 cis-10-pentadecanoid acid, C16:0 palmitic acid, C16:1 palmitoleic acid, C17:0 heptadecanoic acid, C17:1 cis-10-heptadecanoid acid, C18:0 stearic acid, C18:1n9 ct oleic acid, C18:1 vaccinic acid, C18:2n6c linoleic acid, C18:2n6t linoleic acid, C18:3n6 gamma linoleic acid, C18:3n3 linolenic acid, C18:4 stearidonic acid, C20:0 arachidic acid, C22:1/C20:1 cis-11-eicosanic acid, C20:2 Cis-11-eicodienoic acid, C20:3n6 eicosatrienoic acid, C20:4n6 arachidonic acid, C20:3n3 cis-11-eicosatrienoic acid, C20:5n3 eicosapentaenoic acid, C22:0 behenic acid, C22:1n9 13 erucic acid, C22:2 cis-docodienoic acid, C23:0 tricosanoic acid, C22:4n6 docosatetraenoate, C22:5w3 docosapentaenate, C24:0 lignoceric acid, C22:6n3 docosahexaenoic acid, C24:1 nervonic acid.

### 2.3. Interleukin-1 Beta and Interleukin-1 Receptor Antagonist Analysis

Serum IL-1β level was determined using ELISA kit from SunRed Biotechnology Company (Shanghai, China) with detection limit of 0.028 pg/mL and the standard curve in the range of 0.15–4.8 pg/mL. Serum IL-1Ra level was determined using ELISA kit from R&D Systems Inc. (Minneapolis, MN, USA) with detection limit of 18.3 pg/mL and the standard curve in the range of 31.2–2000 pg/mL. The average intra-assay coefficients of variation (intra-assay CV) for the used enzyme immunoassay tests ELISA were <8%. All samples were analysed in a single assay to avoid inter-assay variability.

### 2.4. Statistical Analysis

Initial Correlation Analysis was performed with the use of Spearman’s rank correlation coefficients (ρ/rho) were calculated to assess bivariate relationships between individual fatty acids, IL-1β and IL1-Ra levels. The skewness and kurtosis values showed moderate non-normality, so this test was applied. Statistical significance was set at *p* < 0.05. Multivariate analysis with the use of principal component analysis (PCA) to address multicollinearity among fatty acids. The data was standardised prior to analysis. Components were retained based on eigenvalues greater than 1, and Varimax rotation was applied to enhance interpretability of the components. Partial correlation analysis was performed to evaluate the independence of relationships and confounding assessment. The relationships between fatty acids, IL-1β and lL1-Ra were reassessed while controlling for other fatty acids. Changes in correlation coefficients exceeding 0.1 were considered indicative of potential confounding. Multiple linear regression was conducted using the PCA components as predictors. Model assumptions were verified. R-squared and adjusted R-squared values were calculated to assess the proportion of variance explained by the model. Comparison between groups (study vs. control) employed the two-tailed Mann–Whitney U test. Individual component contributions were assessed using t-statistics and their associated *p*-values. All statistical analyses were performed in Python (version 3.x) using the scipy.stats (version 1.16.2), sklearn (version 1.7.2), and stats models packages (version 0.14.0).

## 3. Results

The results for IL-1β were as follows (mean ± SD, n = 73): 1.31 ± 1.54 pg/mL, while IL-1Ra levels were 810.78 ± 691.02 pg/mL. Positive correlations were observed between certain eicosanoids and IL-1Ra, whereas no significant associations were found with IL-1β. Notably, the direction of correlations for IL-1β was opposite to those detected for IL-1Ra. The demographic characteristic is presented in Table 1.

Spearman’s analysis identified several free fatty acids (FFAs) that correlated with IL-1β levels (Table 2): C15:0 pentadecanoic acid (rho = 0.488), C15:1 cis-10 pentadecanoic acid (rho = 0.473), C17:1 cis-10 heptadecanoic acid (rho = 0.411), C18:0 stearic acid (rho = 0.302), C24:0 lignoceric acid (rho = −0.280), C24:1 nervonic acid (rho = −0.276), C18:2n6t linoleic acid (rho = −0.272), C17:0 heptadecanoic acid (rho = 0.241), and C13:0 tridecanoic acid (rho = 0.238).

Free fatty acids, that were the most significantly correlated with IL-1β levels were C15:0 pentadecanoid acid, C15:1 cis 10 pentadecanoid acid and C17:1 cis 10 heptadecanoid acid. Their r^2^ values and 95% confidence intervals were r^2^ = 0.238, 95% CI: 0.2783, 0.6556; r^2^ = 0.224, 95% CI: 0.2571, 0.6547 and r^2^ = 0.169, 95% CI: 0.2024, 0.5908, respectively.

**Figure 1:**
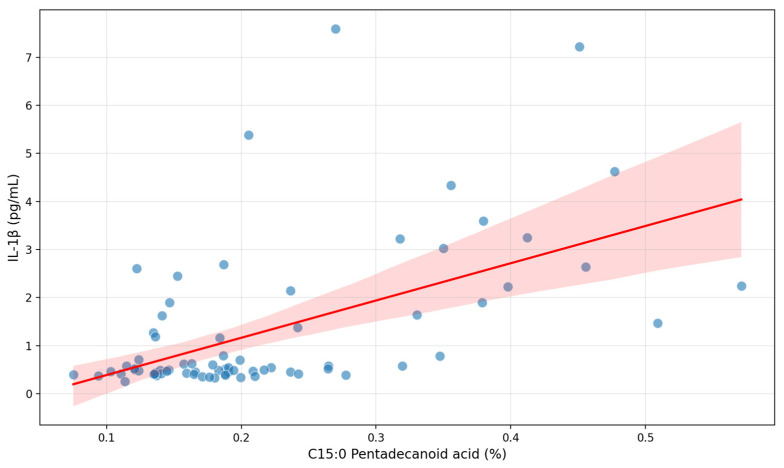
Correlation between C15:0 pentadecanoid acid and serum IL-1β in patients with early subacute phase of stroke.

After multivariate analysis C15:0 pentadecanoid acid remained independently significant (Figure 1) with adjusted r^2^ = 0.273, *p* = 0.005, coefficient 6.19 (95% CI: 1.94; 10.44).

**Figure 2:**
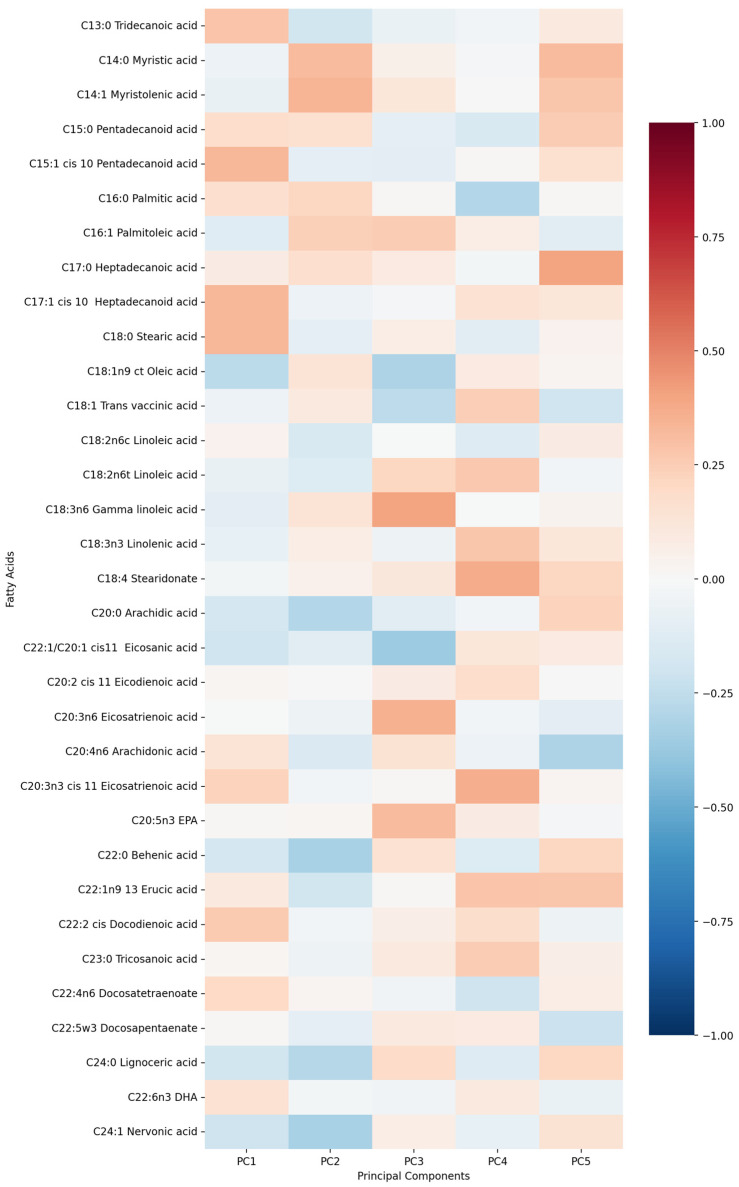
PCA analysis of free fatty acids in regard to IL-1β in patients with early subacute phase of stroke.

**Figure 3:**
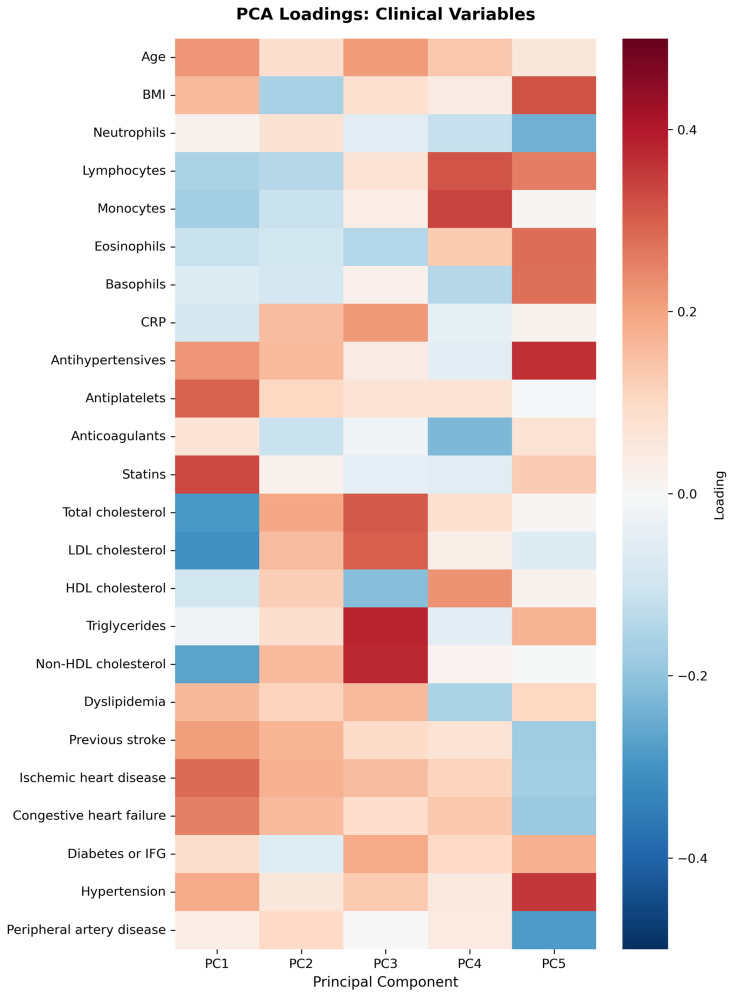
PCA analysis of clinical variables in regard to IL-1β in patients with early subacute phase of stroke.

First five components of PCA analysis of free fatty acids and clinical variables in regard to the impact on IL-1β is presented in Figure 2 and Figure 3. Their combined variance is 44.83%. The most significant variables for each of first five components and their percentage is as follows:

1. PC1 (13.13%)—odd-chain fatty acids axis: C18:0 stearic acid, C17:1 heptadecanoid acid and C15:1 pentadecanoid acid.
2. PC2 (9.74%)—cardiovascular medication/disease axis: statins, IHD and antiplatelets.
3. PC3 (9.01%)—very long-chain fatty acids axis: C20:0 arachidic acid, C24:1 nervonic acid and C22:0 behenic acid.
4. PC4 (7.20%)—omega-3/omega-6 fatty acids axis: C18:3n6 gamma linoleic acid, C20:5n3 EPA and triglycerides.
5. PC5 (5.75%)—monounsaturated fatty acids axis: C18:1n9 oleic acid, C22:1 eicosanic acid and C18:4 stearidonate.

Table 3 presents Spearman’s correlations between free fatty acids and IL1-Ra. Significant positive correlations were observed for C15:1 cis-10-pentadecanoid acid, C18:2n6t linoleic acid, C24:1 nervonic acid, C22:1n9 13 erucic acid, C18:3n6 gammalinoleic acid, C18:4 stearidonate, C24:0 lignoceric acid, C18:2n6c linoleic acid, C22:4n6 docosatetraenoate and C22:0 behenic acid.

**Figure 4:**
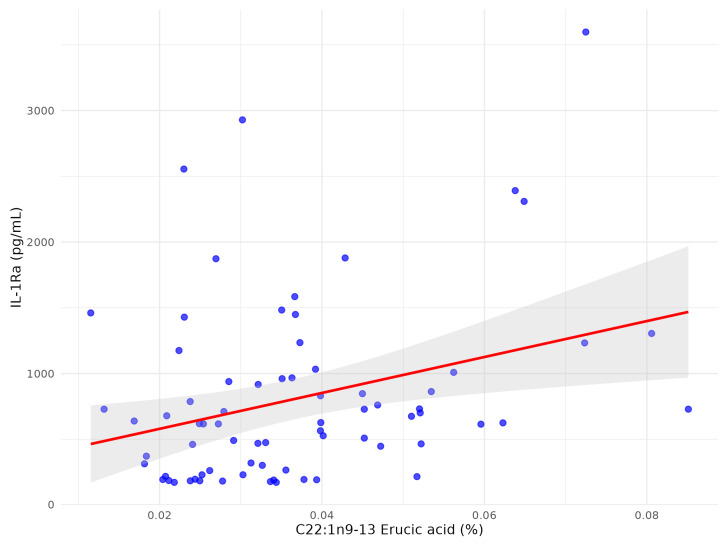
The scatter plot between C22:1n9 13 erucic acid and serum IL1-Ra after multivariate analysis in patients with early subacute phase of stroke.

**Figure 5:**
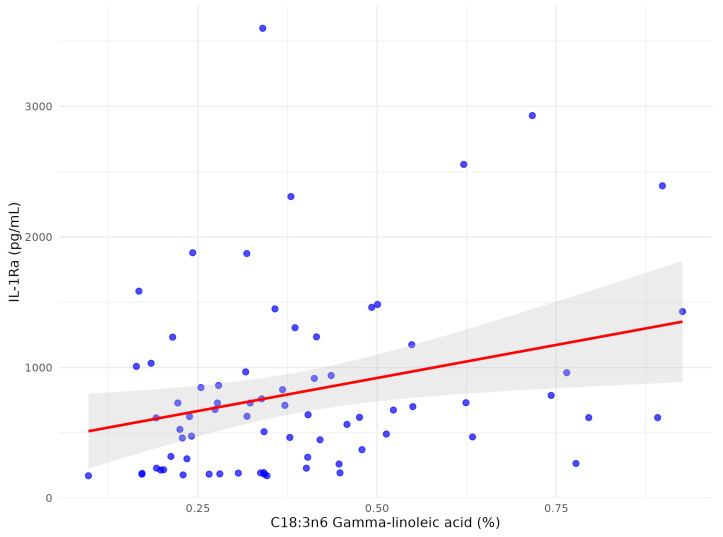
The scatter plot between C18:3n6 gamma-linoleic acid and serum IL1-Ra after multivariate analysis in patients with early subacute phase of stroke.

Following multivariate analysis adjusted for clinical confounders and other FFAs only two FFA remained significantly associated with IL-1Ra: C22:1n9 13 erucic acid (Figure 4) and C18:3n6 gamma-linoleic acid (Figure 5), while C22:4n6 docosatetraenoate was near significantly associated with *p* = 0.055 (Table 4).

**Figure 6:**
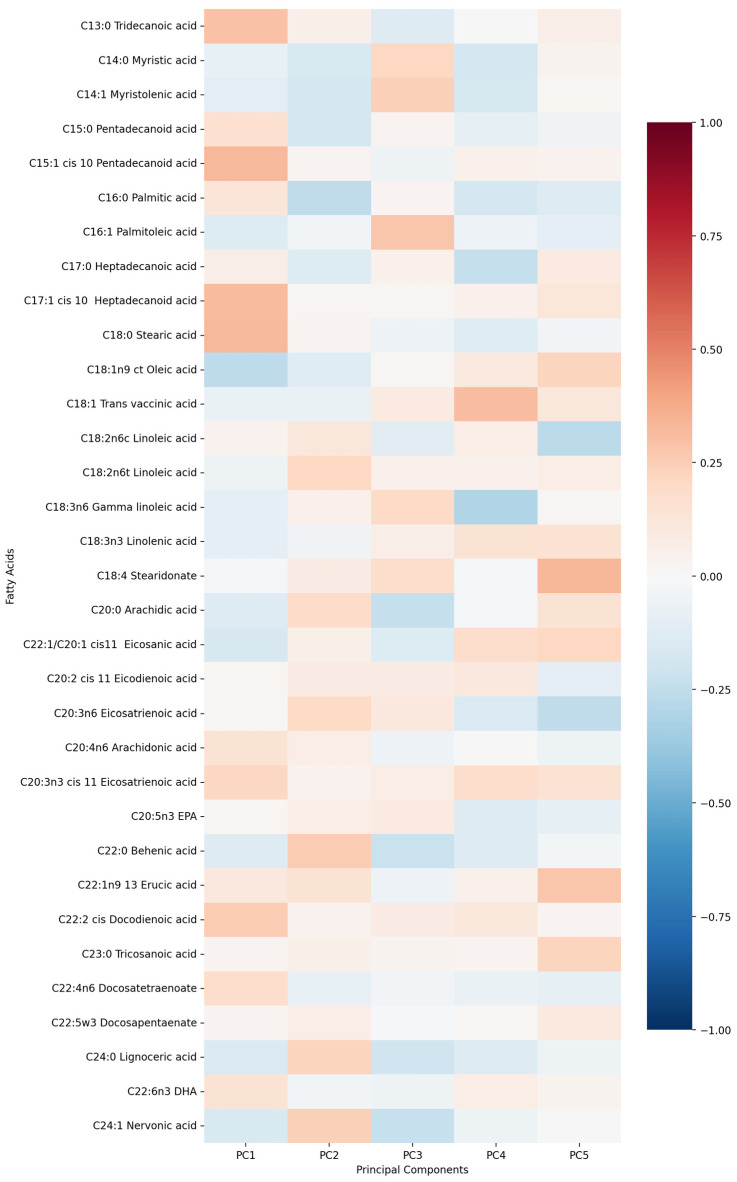
PCA analysis of free fatty acids in regard to serum IL1-Ra in patients with early subacute phase of stroke.

**Figure 7:**
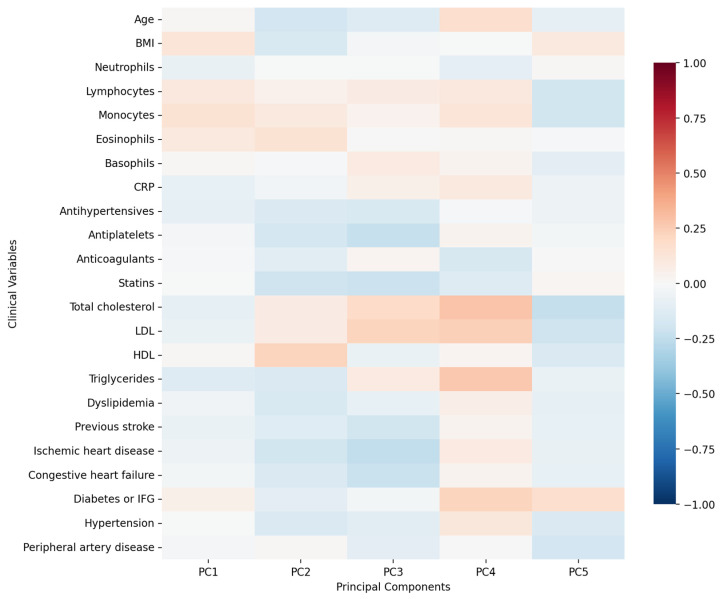
PCA analysis of clinical variables in regard to serum IL1-Ra in patients with early subacute phase of stroke.

First five components of PCA analysis of free fatty acids and clinical variables in regard to the impact on IL1-Ra is presented in Figure 6 and Figure 7. Their combined variance is 43.5%. The most significant variables for each of first five components and their percentage is as follows:

1. PC1 (12.29%)—inflammation–lipids axis: C15:1 cis 10 pentadecanoid acid, C18:0 stearic acid, C17:1 cis 10 heptadecanoid acid.
2. PC2 (9.94%)—lipids–metabolic axis: HDL, total cholesterol, LDL.
3. PC3 (9.12%)—cell counts axis: neutrophils, lymphocytes, monocytes.
4. PC4 (6.56%)—medications/comorbidities axis: statins, antiplatelets, anticoagulants.
5. PC5 (5.95%)—fatty acids axis: C22:1n9 13 erucic acid, C18:2n6c linoleic acid, C18:4 stearidonate.

Comparison of free fatty acids between stroke patients and healthy controls is presented in Table 5.

**Figure 8:**
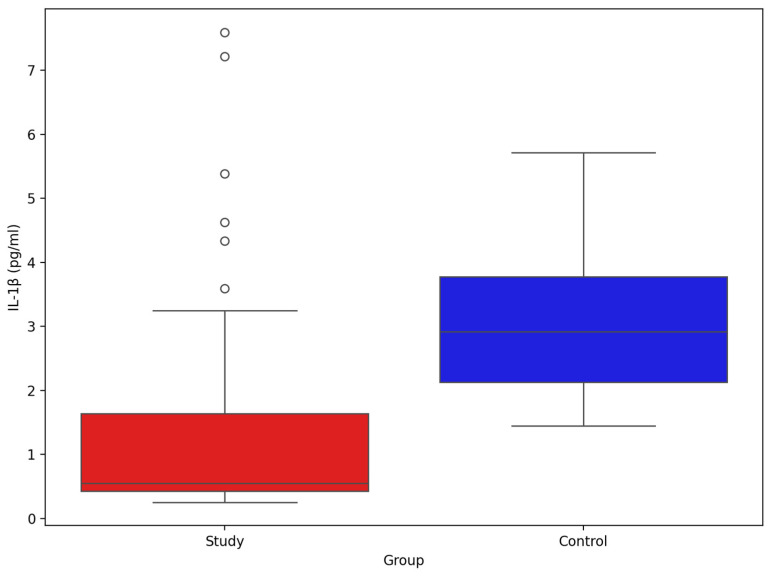
Comparison of serum IL-1β between stroke patients (n = 73) and healthy controls (n = 33).

The comparison of IL-1β between study group and control group (Figure 8) showed significant difference between groups (mean and SD): 1.31 pg/mL (1.54), 3.04 pg/mL (1.20), *p*-value Mann–Whitney U test < 0.001, respectively.

**Figure 9:**
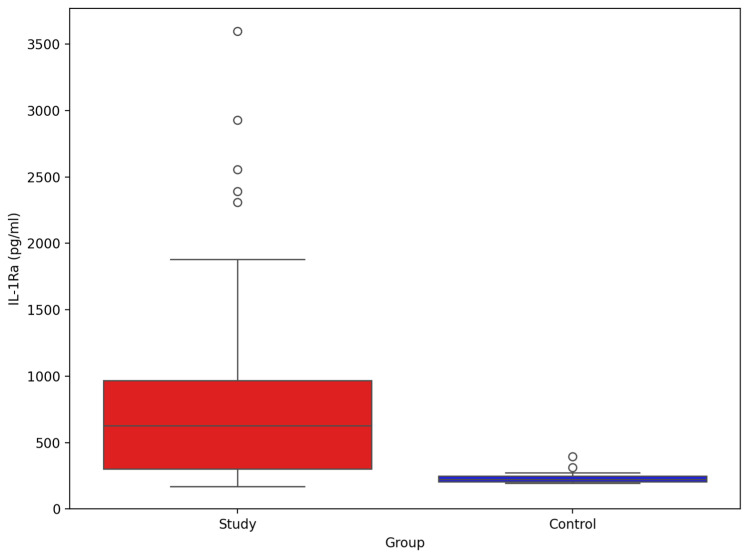
Comparison of serum IL-Ra between stroke patients (n = 73) and healthy controls (n = 33).

The comparison of IL-1β between study group and control group (Figure 9) showed significant difference between groups (mean and SD): 810.78 pg/mL (691.02), 233.24 pg/mL (39.64), *p*-value Mann–Whitney U test < 0.001, respectively.

## 4. Discussion

In patients in the early subacute phase of stroke, we observed an inverse correlation between C24:0 lignoceric acid and IL-1β and a positive correlation with IL-1Ra. Lignoceric acid is a very-long-chain saturated fatty acid (VLCSFA) whose metabolism is linked to PPAR signalling and very-long-chain acyl-CoA synthases. Population studies have associated higher circulating VLCSFAs with more favourable metabolic and cardiovascular profiles. Plasma C24:0 lignoceric acid correlates positively with HDL cholesterol and inversely with the incidence of type 2 diabetes, ischaemic heart disease, sudden cardiac arrest, and triglyceride concentrations. Elevated VLCSFAs have also been linked to a lower incidence of unhealthy ageing [19,20,21,22,23]. However, their functional role in post-ischaemic inflammation remains uncertain. Our results may confirm the anti-inflammatory properties of C24:0 lignoceric acid and its potential beneficial effects on the risk of cerebrovascular events. Given that laboratory studies show certain VLCFAs can induce IL-1β via JNK activation in macrophages, the inverse correlation of C24:0 with IL-1β and its positive correlation with IL-1Ra in our cohort likely reflect context-dependent biology rather than a uniform anti- or pro-inflammatory effect [24]. These associations should therefore be considered hypothesis-generating rather than evidence of clinical benefit.

Among fatty acids, the strongest positive correlations with IL-1β in our early subacute phase of stroke cohort were observed for C15:0 pentadecanoic acid and C15:1 cis-10 pentadecanoic acid, with C15:0 pentadecanoid acid remaining significant after multivariate analysis. Prior work has linked odd-chain species to autoimmune and inflammatory phenotypes [25]. In our data, C15:1 cis-10 pentadecanoic acid was positively associated with IL-1β before multivariate adjustment and inversely associated with IL1-Ra, consistent with opposing directions of these interleukins, yet still observational.

C24:1 nervonic acid showed an inverse correlation with IL-1β in stroke patients. Nervonic acid is a very long-chain saturated fatty acid (VLCSFA) and plays a crucial role as a component of nervonyl sphingolipids, which are key constituents of myelin. Its role in remyelination and metabolic health have been reported [26,27]. Together with erucic acid, nervonic acid correlated inversely with IL-1β, while erucic acid correlated positively with IL-1Ra, a pattern consistent with anti-inflammatory associations [28]. Due to the type of our study the causative role cannot be proven.

C18:2n6 trans-linoleic acid (linoelaidic acid) also showed an inverse correlation with IL-1β and a positive correlation with IL-1Ra. As a trans isomer prevalent in hardened fats, this fatty acid is generally associated with adverse health outcomes, including cancer, diabetes, ischaemic heart disease, chronic heart failure and stroke [29,30,31,32,33,34]. Any apparent anti-inflammatory association in our dataset should be interpreted cautiously and may reflect post-ischaemic lipid remodelling rather than a beneficial effect.

C22:4n6 docosatetraenoate (adrenic acid, AdA) correlated inversely with IL-1Ra and was near-significant after multivariate adjustment (*p* = 0.055). AdA, an n-6 fatty acid involved in membrane biology and beta-oxidation, has been linked to pro-inflammatory signalling and adverse cardio-metabolic phenotypes, including higher CHD and large-artery stroke risk [35,36]. The modest association observed here requires replication.

C22:1n9 erucic acid showed the strongest positive correlation with IL-1Ra after multivariate analysis. Although erucic acid has been associated with potential cardiotoxicity, experimental studies indicate anti-inflammatory, anti-tumour, and neuroprotective properties, potentially via peroxisome proliferator-activated receptors (PPARs) activation and suppression of p38 MAPK and NF-κB pathways [37,38]. Available data do not show an increased stroke risk with erucic acid intake [39]. The clinical significance of circulating erucic acid in stroke remains uncertain.

C18:3n6 Gamma-linoleic acid (GLA) was positively associated with IL1-Ra in a multivariate analysis. GLA is converted to dihomogammalinolenic acid (DGLA), a substrate for anti-inflammatory eicosanoids, and has been linked to cardiometabolic and neurocognitive phenotypes [40,41,42]. In our data, the GLA–IL-1Ra association is compatible with a compensatory or pro-resolving response but remains observational.

Principal component analysis suggested an odd-chain/SFA axis aligning with IL-1β and a lipid–metabolic/medication axis aligning with IL-1Ra. A parsimonious mechanistic framework is that selected saturated/odd-chain FFAs can prime TLR4/MyD88 and NLRP3/caspase-1 signalling, favouring IL-1β maturation, whereas certain VLCSFAs, monounsaturated fats, and PUFAs may engage PPAR and pro-resolving pathways, favouring IL-1Ra induction. These links are consistent with in vitro and animal data but cannot be confirmed by our cross-sectional dataset [4,7,43,44,45].

Compared with controls, stroke patients exhibited lower circulating IL-1β and higher IL-1Ra. In acute ischaemic stroke, IL-1β rapidly promotes leukocyte recruitment and blood–brain barrier (BBB) dysfunction, whereas IL-1Ra rises as an endogenous brake on IL-1 receptor activation. Sampling at day 7 likely captured a transition from early pro-inflammatory activation to a sustained counter-regulatory response, consistent with higher IL-1Ra and lower peripheral IL-1β. This divergence may reflect rapid IL-1β consumption, short-lived systemic peaks relative to longer-lived IL-1Ra, post-stroke immune reprogramming, and sampling timing. At a mechanistic level, FFAs may influence IL-1 biology through (i) TLR4/MyD88-dependent activation of NLRP3/caspase-1 maturation of IL-1β by specific saturated FFAs, and (ii) PPAR-linked signalling by very-long-chain/unsaturated FFAs that may favour IL-1Ra induction. Conversely, IL-1 signalling can remodel lipid metabolism (e.g., by stimulating lipolysis and altering phospholipase activity), providing a plausible bidirectional relationship [46,47,48,49]. Additional processes—such as IL-1β-induced glycolytic shifts, BBB effects that facilitate lipid/cytokine trafficking, and matrix metalloproteinase upregulation—may further couple cytokine and lipid pathways in stroke [50,51]. Adipose-derived mediators, including adiponectin, can also interact with IL-1β to shape eicosanoid output [52]. While these pathways provide biological plausibility for the observed associations and support exploration of IL-1-targeted strategies (e.g., anakinra) alongside metabolic interventions, our findings remain associative [2,48,53,54,55,56].

Interleukin-1 receptor antagonist (IL-1Ra), an endogenous anti-inflammatory cytokine, may have several potential links to free fatty acids (FFAs) in ischemic stroke pathogenesis. IL-1Ra counterbalances IL-1β by acting as a potent anti-inflammatory protein. It is also known to be synthesised following stroke to promote pro-resolving activity and limit the damage caused by ischemia, forming part of the body’s natural response to stroke. The activation of TLR4 on immune cells seems to be a key common factor, triggering the activation of NF-κB and the synthesis of pro-inflammatory cytokines such as IL-1β. In contrast, IL-1Ra inhibits IL-1β, preventing the synthesis of pro-inflammatory molecules. By inhibiting IL-1β, IL-1Ra also helps protect the blood–brain barrier (BBB) from the infiltration of cytokines into the brain parenchyma [57,58]. In response to inflammation, IL-1Ra levels are known to be elevated by FFAs in adipocytes [59]. Fasting increases circulating FFAs and induces the expression of IL-1Ra, promoting anti-inflammatory activity [60]. Lower levels of n-6 fatty acids are significantly associated with higher IL-1Ra levels. Moreover, the ratio of n-6 to n-3 FFAs also influences IL-1Ra concentrations [13]. In an experimental model of stroke, administration of IL-1Ra normalised sPLA2 IIA levels, indicating its ability to counteract the inflammatory response after stroke [2]. Experimental evidence also suggests that IL-1Ra administration reduces lipolysis and circulating FFA levels in metabolic disorders, pointing to a bidirectional relationship where IL-1Ra may mitigate FFA-induced inflammation in stroke. Exogenous IL-1Ra (anakinra) has been shown to reduce infarct size in stroke models [61], although it was found ineffective in reducing brain oedema in patients with intracerebral haemorrhage [62,63]. The potential clinical implications of inhibiting the inflammatory response and enhancing FFA levels via IL-1Ra suggest possible therapeutic options for stroke patients. There is a clear interplay between inflammation and FFAs that may be modulated by IL-1Ra.

Human lipidomic studies after acute brain injury report membrane and plasma lipid remodelling that tracks with inflammation and neuronal damage, aligning with our observation that IL-1Ra associates with specific FFAs [64,65]. Microbiota-derived short-chain fatty acids have also been implicated in neurocognitive outcomes [66]. Broader population and experimental data suggest links between certain saturated/VLCSFA profiles and inflammatory or cognitive phenotypes, and between lipid metabolism and ischaemic brain injury. Nevertheless, longitudinal and mechanistic studies are required to test causality and determine whether specific FFA–cytokine patterns are prognostic or modifiable in stroke [67,68,69].

The main limitations of the study are the small sample size and the fact that samples were collected on day 7. The cross-sectional design with a single time-point (day 7) limits temporal and causal inference. Thus, whether FFAs drive IL-1 signalling or IL-1-driven inflammation and stress hormones drive lipolysis cannot be determined here. Group differences (including age) may confound cytokine and lipid profiles. The study size was modest, and multiple correlations were explored; findings should be viewed as exploratory, with replication warranted. Our sampling at day 7 was chosen to capture the early subacute inflammatory milieu in a standardised manner, but serial profiling (baseline, day 7, later follow-up) would better define trajectories of FFAs and IL-1 axis mediators. Longitudinal sampling was not feasible within the present study and should be interpreted as a limitation. Whether FFAs drive IL-1–mediated inflammation or IL-1–driven lipolysis alters FFA composition cannot be determined from this dataset; both directions remain biologically plausible. The ongoing pro-inflammatory and pro-resolving processes could also affect our results and thus should be acknowledged as a limitation and considered as hypothesis-generating. Given the exploratory nature of this study, *p*-values were not adjusted for multiple comparisons. However, the consistency of findings across multivariate analyses supports their robustness.

## 5. Conclusions

Preclinical studies have demonstrated that anakinra, a recombinant form of IL-1Ra, can reduce the size of stroke lesions, decrease inflammation, and improve BBB permeability. Targeting IL-1β or IL-1Ra could represent an effective therapeutic strategy for addressing the inflammatory processes in ischemic stroke, both during the acute phase and as part of the chronic conditions that contribute to atherosclerosis and increase the risk of stroke [44]. In this prospective cohort of 73 patients in the early subacute phase of ischemic stroke, we identified associations between specific circulating FFAs and key IL-1 family mediators. The odd-chain FFAs, most prominently C15:0 pentadecanoic acid and related C15:1/C17:1 species, were positively associated with IL-1β, whereas very-long-chain and certain monounsaturated/PUFA species, including C24:0 lignoceric acid, C24:1 nervonic acid, C22:1 erucic acid and GLA, showed relationships consistent with anti-inflammatory or compensatory IL-1Ra signalling. Multivariate modelling preserved C15:0 as an independent correlate of IL-1β, while C22:1n9 erucic acid and GLA as independent correlates of IL-1Ra.

Our findings support the concept that lipid metabolism and specific FFA species contribute to post-stroke inflammatory tone and recovery biology, and they underscore IL-1β/IL-1Ra as potential mediators linking lipid perturbations with tissue inflammation. Further lipidomic profiling may help identify patient subgroups most likely to benefit from targeted anti-inflammatory or metabolic interventions.

We recommend follow-up studies including larger, multi-timepoint cohorts, mechanistic studies linking candidate FFAs to NLRP3/IL-1 pathway activation in human tissues or relevant models, and interventional trials that combine lipid-modifying and anti-IL-1 strategies with lipidomic end-points. Such integrated translational efforts could clarify causality and inform personalised anti-inflammatory approaches in stroke care.

## Figures and Tables

**Table 1:** Demographic characteristics of participants.

Characteristic	Value
Age (years, mean ± SD)	60.6 ± 11.9
Sex (Male), n (%)	32 (43.8)
BMI (mean ± SD)	28.7 ± 4.9
Hypertension, n (%)	60 (82.2)
Diabetes/IFG, n (%)	35 (47.9)
Current Smoking, n (%)	25 (34.2)
Dyslipidaemia, n (%)	41 (56.2)
Atrial Fibrillation, n (%)	4 (5.5)
Previous Stroke/TIA, n (%)	5 (6.8)
CRP (mg/L)	2.6 ± 3.7
Total Cholesterol (mg/dL)	196.4 ± 53.0
LDL (mg/dL)	113.4 ± 45
HDL (mg/dL)	52.1 ± 15.2
Triglycerides (mg/dL)	156.1 ± 75.3

**Table 2:** Correlation between serum free fatty acids and IL-1β in patients with early subacute phase of stroke.

Variable	Mean	SD	Spearman Correlation (rho)	*p*-Value
C15:0 pentadecanoid acid	0.2172	0.1076	0.488	<0.001
C15:1 cis-10-pentadecanoid acid	0.0811	0.036	0.4731	<0.001
C17:1 cis-10-heptadecanoid acid	0.0909	0.0349	0.4113	<0.001
C18:0 stearic acid	13.3136	1.9834	0.3019	<0.05
C24:0 lignoceric acid	0.1527	0.0762	−0.2799	<0.05
C24:1 nervonic acid	0.3995	0.2469	−0.2759	<0.05
C18:2n6t linoleic acid	6.1411	1.9309	−0.2718	<0.05
C17:0 heptadecanoic acid	0.302	0.0503	0.2407	<0.05
C13:0 tridecanoic acid	0.3071	0.0916	0.2382	<0.05
C23:0 tricosanoic acid	0.2329	0.1517	−0.2302	0.05
C22:0 behenic acid	0.2252	0.0979	−0.224	0.0568
C18:3n6 gammalinoleic acid	0.3862	0.1921	−0.2215	0.0597
C22:4n6 docosatetraenoate	0.2226	0.1165	0.2149	0.0678
C20:3n6 eicosatrienoic acid	1.2825	0.3089	−0.2141	0.069
C16:1 palmitoleic acid	2.1342	0.7468	−0.2034	0.0844
C22:6n3 docosahexaenoic acid (DHA)	1.7522	0.5305	0.1801	0.1273
C16:0 palmitic acid	26.8018	1.7418	0.1715	0.1469
C22:1n9 13 erucic acid	0.037	0.0156	−0.1684	0.1545
C20:5n3 eicosapentaenoic acid (EPA)	0.6033	0.2581	−0.1549	0.1906
C20:0 arachidic acid	0.2061	0.073	−0.1492	0.2078
C14:1 myristolenic acid	0.0698	0.0377	0.1389	0.2411
C22:2-cis-docodienoic acid	0.0169	0.0109	0.1291	0.2762
C14:0 myristic acid	1.2079	0.3812	0.1281	0.2802
C20:2 cis-11-eicodienoic acid	0.1506	0.0339	−0.1277	0.2816
C18:4 stearidonate	0.0575	0.0273	−0.1161	0.328
C22:1/C20:1 cis11-eicosanic acid	0.1785	0.0687	0.0966	0.4162
C22:5w3 docosapentaenate	0.4603	0.2315	−0.0936	0.4311
C18:3n3 linolenic acid	0.5037	0.1586	−0.0817	0.4918
C20:3n3 cis-11-eicosatrienoic acid	0.0309	0.0143	0.0471	0.6921
C20:4n6 arachidonic acid	6.3053	1.3142	0.0341	0.7746
C18:1 vaccinic acid	1.9785	0.3506	0.0143	0.9046
C18:2n6c linoleic acid	11.5384	2.3326	0.0087	0.9419
C18:1n9 ct oleic acid	22.5909	3.7134	−0.0071	0.9522

**Table 3:** Correlation between serum free fatty acids and IL1-Ra in patients with early subacute phase of stroke.

Free Fatty Acid	Mean	SD	Spearman Correlation (rho)	*p*-Value
C15:1 cis-10-pentadecanoid acid	0.081	0.036	−0.357	<0.005
C18:2n6t linoleic acid	6.141	1.931	0.341	<0.005
C24:1 nervonic acid	0.4	0.247	0.302	<0.05
C22:1n9 13 erucic acid	0.037	0.016	0.299	<0.05
C18:3n6 gammalinoleic acid	0.386	0.192	0.277	<0.05
C18:4 stearidonate	0.057	0.027	0.255	<0.05
C24:0 lignoceric acid	0.153	0.076	0.254	<0.05
C18:2n6c linoleic acid	11.538	2.333	−0.254	<0.05
C22:4n6 docosatetraenoate	0.223	0.116	−0.241	<0.05
C22:0 behenic acid	0.225	0.098	0.236	<0.05
C17:1 cis-10-heptadecanoid acid	0.091	0.035	−0.228	0.052
C15:0 pentadecanoid acid	0.217	0.108	−0.209	0.077
C22:1/C20:1 cis-11-eicosanic acid	0.179	0.069	−0.205	0.082
C20:0 arachidic acid	0.206	0.073	0.202	0.086
C20:5n3 eicosapentaenoic acid (EPA)	0.603	0.258	0.193	0.102
C20:2 cis-11-eicodienoic acid	0.151	0.034	−0.176	0.136
C18:0 stearic acid	13.314	1.983	−0.166	0.16
C23:0 tricosanoic acid	0.233	0.152	0.162	0.17
C13:0 tridecanoic acid	0.307	0.092	−0.144	0.225
C18:1 trans vaccinic acid	1.978	0.351	−0.143	0.228
C16:1 palmitoleic acid	2.134	0.747	0.131	0.27
C16:0 palmitic acid	26.802	1.742	−0.106	0.373
C18:3n3 linolenic acid	0.504	0.159	0.095	0.426
C22:2 cis-docodienoic acid	0.017	0.011	−0.077	0.515
C14:1 myristolenic acid	0.07	0.038	0.072	0.544
C22:6n3 docosahexaenoic acid (DHA)	1.752	0.531	−0.057	0.631
C20:3n6 eicosatrienoic acid	1.283	0.309	0.043	0.716
C20:3n3 cis-11-eicosatrienoic acid	0.031	0.014	−0.043	0.716
C22:5w3 docosapentaenate	0.46	0.231	0.04	0.738
C20:4n6 arachidonic acid	6.305	1.314	−0.038	0.751
C17:0 heptadecanoic acid	0.302	0.05	−0.035	0.769
C14:0 myristic acid	1.208	0.381	0.013	0.914
C18:1n9 ct oleic acid	22.591	3.713	−0.007	0.956

**Table 4:** Association between serum IL1-Ra after multivariate analysis for confounding factors in patients with early subacute phase of stroke.

Free Fatty Acid	Spearman (rho)	Beta Coefficient (95% CI)	*R* ^2^	*p*-Value
C18:3n6 gamma-linoleic acid	0.277	1343 (−3114; 36.72)	0.0581	<0.05
C22:1n9 13 erucic acid	0.299	20,612.8 (8710; 32,515)	0.1124	<0.05
C22:4n6 docosatetraenoate	−0.241	1538 (−3114; 36.72)	0.0358	0.055

**Table 5:** Comparison of free fatty acids between stroke patients and healthy controls.

Fatty Acid	Study Group (n = 73): Mean (SD)	Control Group (n = 33): Mean (SD)	*p*-Value (Mann–Whitney U Test)
C15:1 cis-10-pentadecanoid acid	0.08 (0.04)	0.31 (0.15)	<0.01
C18:2n6t linoleic acid	6.09 (1.92)	16.22 (6.21)	<0.01
C24:1 nervonic acid	0.4 (0.25)	0.16 (0.21)	<0.01
C22:1n9 13 erucic acid	0.04 (0.02)	0.07 (0.03)	<0.01
C18:3n6 gammalinoleic acid	0.39 (0.19)	0.6 (0.31)	<0.01
C18:4 stearidonate	0.06 (0.03)	0.12 (0.06)	<0.01
C24:0 lignoceric acid	0.15 (0.08)	0.11 (0.16)	<0.01
C18:2n6c linoleic acid	11.55 (2.36)	30.2 (14.34)	<0.01
C22:4n6 docosatetraenoate	0.22 (0.12)	0.63 (0.36)	<0.01
C22:0 behenic acid	0.23 (0.1)	0.21 (0.2)	<0.01
C17:1 cis-10-heptadecanoid acid	0.09 (0.03)	0.3 (0.13)	<0.01
C15:0 pentadecanoid acid	0.22 (0.11)	0.49 (0.27)	<0.01
C22:1/C20:1 cis-11-eicosanic acid	0.18 (0.07)	0.43 (0.22)	<0.01
C20:0 arachidic acid	0.21 (0.07)	0.35 (0.19)	<0.01
C20:5n3 eicosapentaenoic acid (EPA)	0.61 (0.26)	2.79 (2.66)	<0.01
C20:2 cis-11-eicodienoic acid	0.15 (0.03)	0.41 (0.19)	<0.01
C18:0 stearic acid	13.36 (1.94)	33.81 (13.16)	<0.01
C23:0 tricosanoic acid	0.23 (0.14)	0.37 (0.45)	<0.01
C13:0 tridecanoic acid	0.31 (0.09)	0.88 (0.45)	<0.01
C18:1 trans vaccinic acid	1.98 (0.35)	3.99 (1.84)	<0.01
C16:1 palmitoleic acid	2.15 (0.75)	4.02 (2.23)	<0.01
C16:0 palmitic acid	26.8 (1.76)	60.19 (23.56)	<0.01
C18:3n3 linolenic acid	0.5 (0.15)	1.68 (0.88)	<0.01
C22:2 cis-docodienoic acid	0.02 (0.01)	0.04 (0.02)	<0.01
C14:1 myristolenic acid	0.07 (0.04)	0.16 (0.13)	<0.01
C22:6n3 docosahexaenoic acid (DHA)	1.75 (0.54)	5.48 (2.88)	<0.01
C20:3n6 eicosatrienoic acid	1.29 (0.31)	2.8 (1.09)	<0.01
C20:3n3 cis-11-eicosatrienoic acid	0.03 (0.01)	0.1 (0.04)	<0.01
C22:5w3 docosapentaenate	0.46 (0.23)	1.35 (0.58)	<0.01
C20:4n6 arachidonic acid	6.29 (1.33)	16.39 (7.3)	<0.01
C17:0 heptadecanoic acid	0.3 (0.05)	0.77 (0.35)	<0.01
C14:0 myristic acid	1.22 (0.38)	2.7 (1.67)	<0.01
C18:1n9 ct oleic acid	22.55 (3.74)	42.84 (19.92)	<0.01

## Data Availability

The raw data supporting the conclusions of this article will be made available by the authors on request.
